# Supplementary material for: Risk factors for the development of hospital-acquired pediatric venous thromboembolism—Dealing with potentially causal and confounding risk factors using a directed acyclic graph (DAG) analysis
Source: PLoS One. 2020 Nov 13;15(11):e0242311. doi: 10.1371/journal.pone.0242311 (PMC7665816; doi:10.1371/journal.pone.0242311)
Supplement: S1 Table — (DOCX) [file pone.0242311.s001.docx]

| **S1 Table. DAG flowchart code.** |
| --- |
| ag {  "Autoimmune/inflammatory disease" [pos="0.284,0.699"]  "Family history of thrombosis (1st degree)" [latent,pos="0.276,0.932"]  "Heart failure" [exposure,pos="0.291,0.812"]  "Hematologic Malignancies" [pos="0.229,0.700"]  "ICU admission" [exposure,pos="0.152,0.804"]  "L-asparaginase" [pos="0.260,0.690"]  "Length of stay (LOS)" [exposure,pos="0.142,0.972"]  "Liver failure" [exposure,pos="0.168,0.722"]  "Local Trauma" [pos="0.191,0.708"]  "Mechanical ventilation" [exposure,pos="0.139,0.724"]  "Nephrotic syndrome" [pos="0.171,0.931"]  "Venous thromboembolism (VTE)" [outcome,pos="0.217,0.984"]  Catheter [exposure,pos="0.272,0.833"]  Corticosteroids [pos="0.278,0.768"]  Dehydration [latent,pos="0.287,0.898"]  Immobilization [pos="0.137,0.873"]  Infection [pos="0.208,0.801"]  Obesity [exposure,pos="0.294,0.862"]  Surgery [pos="0.215,0.732"]  Thrombophilia [latent,pos="0.264,0.971"]  "Autoimmune/inflammatory disease" -> "Venous thromboembolism (VTE)"  "Autoimmune/inflammatory disease" -> Corticosteroids  "Family history of thrombosis (1st degree)" -> "Venous thromboembolism (VTE)"  "Family history of thrombosis (1st degree)" -> Thrombophilia  "Heart failure" -> "Venous thromboembolism (VTE)"  "Hematologic Malignancies" -> "L-asparaginase"  "Hematologic Malignancies" -> "Venous thromboembolism (VTE)"  "Hematologic Malignancies" -> Catheter  "Hematologic Malignancies" -> Corticosteroids  "Hematologic Malignancies" -> Infection  "ICU admission" -> "Length of stay (LOS)"  "ICU admission" -> "Venous thromboembolism (VTE)"  "ICU admission" -> Catheter  "ICU admission" -> Immobilization  "ICU admission" -> Infection  "L-asparaginase" -> "Venous thromboembolism (VTE)"  "L-asparaginase" -> Catheter  "Length of stay (LOS)" -> "Venous thromboembolism (VTE)"  "Length of stay (LOS)" -> Immobilization  "Liver failure" -> "Venous thromboembolism (VTE)"  "Local Trauma" -> "Length of stay (LOS)"  "Local Trauma" -> "Venous thromboembolism (VTE)"  "Local Trauma" -> Immobilization  "Mechanical ventilation" -> "ICU admission"  "Mechanical ventilation" -> "Venous thromboembolism (VTE)"  "Mechanical ventilation" -> Immobilization  "Mechanical ventilation" -> Infection  "Nephrotic syndrome" -> "Length of stay (LOS)"  "Nephrotic syndrome" -> "Venous thromboembolism (VTE)"  "Nephrotic syndrome" -> Catheter  "Nephrotic syndrome" -> Corticosteroids  "Nephrotic syndrome" -> Infection  Catheter -> "Venous thromboembolism (VTE)"  Catheter -> Infection  Corticosteroids -> "Venous thromboembolism (VTE)"  Corticosteroids -> Infection  Dehydration -> "Venous thromboembolism (VTE)"  Immobilization -> "Venous thromboembolism (VTE)"  Infection -> "Length of stay (LOS)"  Infection -> "Venous thromboembolism (VTE)"  Infection -> Dehydration  Obesity -> "Venous thromboembolism (VTE)"  Surgery -> "Length of stay (LOS)"  Surgery -> "Venous thromboembolism (VTE)"  Surgery -> Immobilization  Thrombophilia -> "Venous thromboembolism (VTE)"  } |
