# Supplementary material for: Risk factors for the development of hospital-acquired pediatric venous thromboembolism—Dealing with potentially causal and confounding risk factors using a directed acyclic graph (DAG) analysis
Source: PLoS One. 2020 Nov 13;15(11):e0242311. doi: 10.1371/journal.pone.0242311 (PMC7665816; doi:10.1371/journal.pone.0242311)
Supplement: S2 Table — (DOCX) [file pone.0242311.s002.docx]

| **S2 Table. Conditional independence and minimal sufficient adjustment proposed by the DAG model.** |
| --- |
| The model implies the following conditional independences:  • Autoimmune/inflammatory disease ⊥ Heart failure  • Autoimmune/inflammatory disease ⊥ Hematologic Malignancies  • Autoimmune/inflammatory disease ⊥ ICU admission  • Autoimmune/inflammatory disease ⊥ L-asparaginase  • Autoimmune/inflammatory disease ⊥ Length of stay (LOS) \| ICU admission, Infection, Nephrotic syndrome  • Autoimmune/inflammatory disease ⊥ Length of stay (LOS) \| Corticosteroids, Hematologic Malignancies, Nephrotic syndrome  • Autoimmune/inflammatory disease ⊥ Liver failure  • Autoimmune/inflammatory disease ⊥ Local Trauma  • Autoimmune/inflammatory disease ⊥ Mechanical ventilation  • Autoimmune/inflammatory disease ⊥ Nephrotic syndrome  • Autoimmune/inflammatory disease ⊥ Catheter  • Autoimmune/inflammatory disease ⊥ Immobilization \| ICU admission, Length of stay (LOS), Local Trauma, Mechanical ventilation, Surgery  • Autoimmune/inflammatory disease ⊥ Immobilization \| ICU admission, Infection, Mechanical ventilation, Nephrotic syndrome  • Autoimmune/inflammatory disease ⊥ Immobilization \| Corticosteroids, Hematologic Malignancies, Nephrotic syndrome  • Autoimmune/inflammatory disease ⊥ Infection \| Corticosteroids, Hematologic Malignancies, Nephrotic syndrome  • Autoimmune/inflammatory disease ⊥ Obesity  • Autoimmune/inflammatory disease ⊥ Surgery  • Heart failure ⊥ Hematologic Malignancies  • Heart failure ⊥ ICU admission  • Heart failure ⊥ L-asparaginase  • Heart failure ⊥ Length of stay (LOS)  • Heart failure ⊥ Liver failure  • Heart failure ⊥ Local Trauma  • Heart failure ⊥ Mechanical ventilation  • Heart failure ⊥ Nephrotic syndrome  • Heart failure ⊥ Catheter  • Heart failure ⊥ Corticosteroids  • Heart failure ⊥ Immobilization  • Heart failure ⊥ Infection  • Heart failure ⊥ Obesity  • Heart failure ⊥ Surgery  • Hematologic Malignancies ⊥ ICU admission  • Hematologic Malignancies ⊥ Length of stay (LOS) \| ICU admission, Infection, Nephrotic syndrome  • Hematologic Malignancies ⊥ Liver failure  • Hematologic Malignancies ⊥ Local Trauma  • Hematologic Malignancies ⊥ Mechanical ventilation  • Hematologic Malignancies ⊥ Nephrotic syndrome  • Hematologic Malignancies ⊥ Immobilization \| ICU admission, Length of stay (LOS), Local Trauma, Mechanical ventilation, Surgery  • Hematologic Malignancies ⊥ Immobilization \| ICU admission, Infection, Mechanical ventilation, Nephrotic syndrome  • Hematologic Malignancies ⊥ Obesity  • Hematologic Malignancies ⊥ Surgery  • ICU admission ⊥ L-asparaginase  • ICU admission ⊥ Liver failure  • ICU admission ⊥ Local Trauma  • ICU admission ⊥ Nephrotic syndrome  • ICU admission ⊥ Corticosteroids  • ICU admission ⊥ Obesity  • ICU admission ⊥ Surgery  • L-asparaginase ⊥ Length of stay (LOS) \| ICU admission, Infection, Nephrotic syndrome  • L-asparaginase ⊥ Length of stay (LOS) \| Catheter, Hematologic Malignancies, ICU admission, Nephrotic syndrome  • L-asparaginase ⊥ Liver failure  • L-asparaginase ⊥ Local Trauma  • L-asparaginase ⊥ Mechanical ventilation  • L-asparaginase ⊥ Nephrotic syndrome  • L-asparaginase ⊥ Corticosteroids \| Hematologic Malignancies  • L-asparaginase ⊥ Immobilization \| ICU admission, Length of stay (LOS), Local Trauma, Mechanical ventilation, Surgery  • L-asparaginase ⊥ Immobilization \| ICU admission, Infection, Mechanical ventilation, Nephrotic syndrome  • L-asparaginase ⊥ Immobilization \| Catheter, Hematologic Malignancies, ICU admission, Nephrotic syndrome  • L-asparaginase ⊥ Infection \| Catheter, Hematologic Malignancies, ICU admission, Nephrotic syndrome  • L-asparaginase ⊥ Obesity  • L-asparaginase ⊥ Surgery  • Length of stay (LOS) ⊥ Liver failure  • Length of stay (LOS) ⊥ Mechanical ventilation \| ICU admission, Infection, Nephrotic syndrome  • Length of stay (LOS) ⊥ Catheter \| ICU admission, Infection, Nephrotic syndrome  • Length of stay (LOS) ⊥ Corticosteroids \| ICU admission, Infection, Nephrotic syndrome  • Length of stay (LOS) ⊥ Obesity  • Liver failure ⊥ Local Trauma  • Liver failure ⊥ Mechanical ventilation  • Liver failure ⊥ Nephrotic syndrome  • Liver failure ⊥ Catheter  • Liver failure ⊥ Corticosteroids  • Liver failure ⊥ Immobilization  • Liver failure ⊥ Infection  • Liver failure ⊥ Obesity  • Liver failure ⊥ Surgery  • Local Trauma ⊥ Mechanical ventilation  • Local Trauma ⊥ Nephrotic syndrome  • Local Trauma ⊥ Catheter  • Local Trauma ⊥ Corticosteroids  • Local Trauma ⊥ Infection  • Local Trauma ⊥ Obesity  • Local Trauma ⊥ Surgery  • Mechanical ventilation ⊥ Nephrotic syndrome  • Mechanical ventilation ⊥ Catheter \| ICU admission  • Mechanical ventilation ⊥ Corticosteroids  • Mechanical ventilation ⊥ Obesity  • Mechanical ventilation ⊥ Surgery  • Nephrotic syndrome ⊥ Immobilization \| ICU admission, Length of stay (LOS), Local Trauma, Mechanical ventilation, Surgery  • Nephrotic syndrome ⊥ Obesity  • Nephrotic syndrome ⊥ Surgery  • Catheter ⊥ Corticosteroids \| Hematologic Malignancies, Nephrotic syndrome  • Catheter ⊥ Immobilization \| ICU admission, Length of stay (LOS), Local Trauma, Mechanical ventilation, Surgery  • Catheter ⊥ Immobilization \| ICU admission, Infection, Mechanical ventilation, Nephrotic syndrome  • Catheter ⊥ Obesity  • Catheter ⊥ Surgery  • Corticosteroids ⊥ Immobilization \| ICU admission, Length of stay (LOS), Local Trauma, Mechanical ventilation, Surgery  • Corticosteroids ⊥ Immobilization \| ICU admission, Infection, Mechanical ventilation, Nephrotic syndrome  • Corticosteroids ⊥ Obesity  • Corticosteroids ⊥ Surgery  • Immobilization ⊥ Infection \| ICU admission, Length of stay (LOS), Local Trauma, Mechanical ventilation, Surgery  • Immobilization ⊥ Obesity  • Infection ⊥ Obesity  • Infection ⊥ Surgery  • Obesity ⊥ Surgery  Minimal sufficient adjustment sets for estimating the direct effect of Heart failure, ICU admission, Length of stay (LOS), Liver failure, Mechanical ventilation, Catheter, Obesity on Venous thromboembolism (VTE):  • Corticosteroids, Hematologic Malignancies, Immobilization, Infection, L-asparaginase, Local Trauma, Nephrotic syndrome, Surgery. |
